# Uncovering the circadian transcriptome of *Nasonia vitripennis*: insights into a non-canonical insect model

**DOI:** 10.1098/rspb.2024.1848

**Published:** 2024-11-27

**Authors:** Eran Tauber

**Affiliations:** ^1^ Department of Evolutionary & Environmental Biology, Institute of Evolution, University of Haifa, Haifa 3498838, Israel

**Keywords:** circadian clock, transcriptomics, *Nasonia*, RNA-seq

## Abstract

The study of the circadian clock has greatly benefited from using *Drosophila* as a model system. Yet accumulating evidence suggests that the fly might not be the canonical insect model. Here, I have analysed the circadian transcriptome of the jewel wasp *Nasonia vitripennis* by using RNA-seq in both constant darkness and constant light (in contrast to flies, the wasps are rhythmic under continuous light). I identify approximately 6% of the transcriptome as cycling under constant conditions, revealing a bimodal distribution of phases and low cycling amplitude. I examine the biological processes under circadian control in *Nasonia*, identifying clock control of functions such as metabolism, light response and a variety of neural processes, drawing comparisons between *Nasonia* and *Drosophila*. Although there was little similarity between cycling genes in *Drosophila* and *Nasonia*, the functions fulfilled by cycling transcripts were similar in both species. Interestingly, of the known *Drosophila* core clock genes, only *Pdp1e*, *shaggy* and *Clock* showed significant cycling in *Nasonia*, highlighting the potential diversity in molecular clock mechanisms across insect species.

## 1. Introduction

The circadian clock regulates fundamental biological processes such as sleep, metabolism and the immune system [1–3], and has implications for a wide range of human diseases. Notable examples of diseases linked to the circadian clock include cancer [4], Alzheimer’s disease [5], cardiovascular disease [6], obesity [7] and depression [8]. A primary output of the clock is circadian regulation of transcription, a trait demonstrated in mammals [9], insects [10], plants [11] and even bacteria [12]. Therefore, analysing transcriptional oscillations in clock-controlled genes (CCGs) is a key step in understanding how the daily rhythms produced by the clock are ultimately linked to behavioural phenotypes.

The genetic mechanisms underlying the animal circadian clock were first elucidated through studies of model animals, primarily the fruit fly *Drosophila*. The first clock gene to be identified, *period* (*per*), was discovered through mapping the genetic basis of *Drosophila* mutants with aberrant locomotor and eclosion rhythms [13]. The discovery of *period* was followed by the discovery of its heterodimeric partner *timeless* (*tim*) [14]. These two genes are joined by a roster of other genes working together to produce robust internal rhythms.

The discoveries made in *Drosophila* have been instrumental in understanding the mechanisms of the circadian clock in mammals [15]. As the principal insect model, *Drosophila* has been used to great effect to model circadian phenomena in humans [16]. However, as circadian research into non-drosophilid insects has advanced, several alternative clock models have been proposed [17], some of which may better model aspects of the mammalian clock than *Drosophila*.

For example, a major difference between the various clock models in insects concerns the light input pathway. The main light input to the clock in *Drosophila* is mediated through CRYPTOCHROME (CRY1), which is activated in response to light [18], binds to and promotes the degradation of TIM [19]*,* ultimately resulting in the degradation of PER [20,21]. In contrast, mammalian-like CRYPTOCHROME (CRY2) is not light-sensitive [17] but is a part of the core transcriptional feedback loop, suppressing its own transcription (and that of *per*) by interfering with the actions of the CLK-CYC heterodimer [22,23]. Mammals also lack a homologue for *timeless*, possessing only a homologue of the *Drosophila* gene *timeout* [24], a gene whose potential role in the clock is less clear and less crucial than that of *timeless* [25,26].

The Lepidoptera harbour both types of *cryptochrome* (*Drosophila*-like *cry1* and mammal-like *cry2*) [27], as well as homologues of *timeless* and *timeout* [28]. The two CRY proteins have been shown to act in a similar way to their *Drosophila* and mammal counterparts; CRY1 functions as a light receptor and CRY2 serves as a transcriptional repressor [29].

Of the major insect orders, the Hymenoptera arguably possesses the most mammalian-like core clock architecture, possessing *cry2* and *timeout* but neither *cry1* nor *timeless* [17,28]. In addition to these molecular similarities, there is evidence that the transcriptional profiles of these genes match more closely the mammalian model than the *Drosophila* model [30]. Light-entrained circadian rhythms have been demonstrated in the Hymenoptera, but the question of light detection in the Hymenopteran clock remains an open one, although the involvement of the blue and green opsin photoreceptors has been recently proposed [31].

*Nasonia vitripennis* is a parasitoid wasp, which as a research model offers advantages over other hymenopterans, including a fully sequenced genome [32], systemic RNAi [33], a robust and well-characterized circadian response [34], a fully functional DNA methylation kit [35], a CRISPR-directed gene editing [36], and a history as a model for photoperiodism [37].

In this study, I advance *Nasonia* as an alternative circadian model by using RNA-seq to profile whole-transcriptome gene expression in the *Nasonia* head. As the *Nasonia* clock free-runs in both constant darkness (DD) and constant light (LL), I profiled both of these conditions to examine how the two circadian transcriptomes differ. Specifically, this study aims to address the following questions. How do gene expression patterns associated with the circadian clock in *Nasonia* differ between DD and LL? What insights can we gain about the molecular mechanisms underlying circadian rhythm maintenance in *Nasonia* under different lighting conditions? My findings, particularly the limited overlap of rhythmic genes between DD and LL, provide new insights into the plasticity of the circadian clock in Nasonia and its response to different lighting environments.

To our knowledge, this is the first circadian RNA-seq study performed in an insect other than *Drosophila* and the first study to profile the circadian transcriptome oscillating under constant light.

## 2. Results

### (a) Sample collection and sequencing

**Figure 1.**
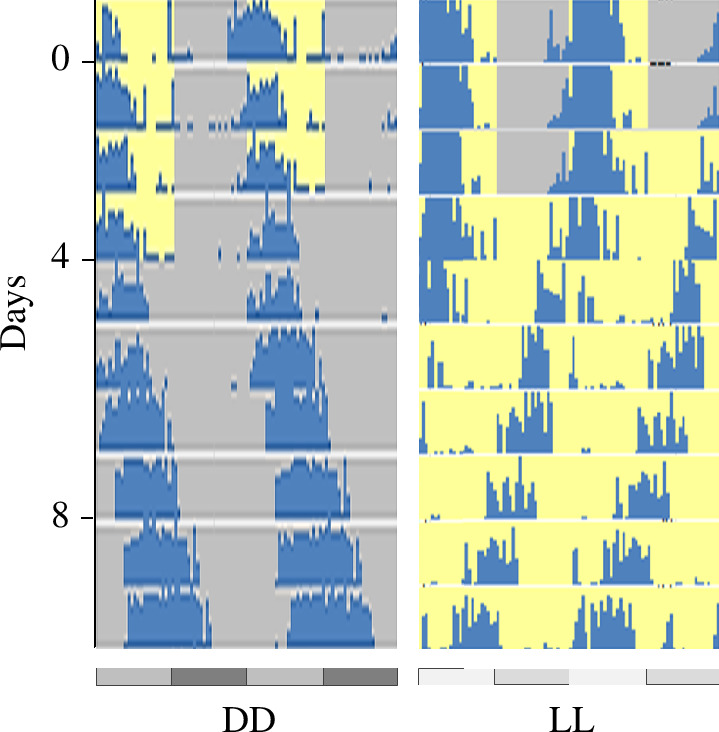
Free-running behavioural rhythm in *Nasonia*. Representative actograms of individual *Nasonia* males in DD (left) and LL (right) are shown. Activity counts were sorted into 30 min bins and plotted in blue. Yellow and grey backgrounds indicate lights on and lights off, respectively. In the DD plot, grey and black bars below the actogram indicate the light and dark fractions, respectively, in which the wasps were originally kept. In the LL plot, the light and dark fractions before the free-run stage are represented by light and darker grey bars, respectively.

Previous studies showed that *N. vitripennis* males and females free-ran in both constant light and constant darkness [34]. *N. vitripennis* females showed considerably less robust rhythms in constant light. Based on these data, I entrained newly enclosed male *N. vitripennis* wasps in two experiments for 4 days and collected wasps every 4 h for 48 h in both DD and LL beginning at 1 h after the wasps were released into free conditions (figure 1). LL and DD were profiled independently (i.e. these two experiments were not run in parallel). For each experiment, groups of 50 male wasp heads were collected at each time point, RNA was extracted and sequenced, and one 75 bp paired-end sample was obtained for each time point in each condition. Reads were mapped to the *N. vitripennis* genome and transcripts were quantified. The free-running periods (19°C) were 25.8 h in DD and 22.4 h in LL.

### (b) Identifying rhythmic transcription

I first performed an unbiased clustering analysis to ascertain the kinds of expression patterns present in each experiment. To this end, Mfuzz [38] was used to carry soft c-means clustering, a method that is less sensitive to biological noise than traditional clustering [39]. After filtering (see §4), 30 clusters were generated for each condition (electronic supplementary material, figures S1 and S2), revealing a variety of potentially rhythmic and non-rhythmic expression trends. Potential asymmetric waveforms were detected in LL (i.e. waveforms with a steeper rise than fall, or vice versa; e.g. electronic supplementary material, figure S2, clusters 22 and 26).

To identify rhythmic transcripts, I used the RAIN algorithm [40], a non-parametric test that treats the rising and falling portions of waves separately, allowing for asymmetric waveforms. The number of rhythmic transcripts at various false discovery thresholds is shown in electronic supplementary material, table S15. At a false discovery rate (FDR) threshold of 0.1 (adjusted *p*‐value, i.e. *q*-value), I identified 1057 rhythmic transcripts in DD and 929 in LL. These transcripts are shown in electronic supplementary material, tables S1 and S2. Seventy-seven per cent of RAIN-identified rhythmic transcripts in DD and 52% of RAIN-identified rhythmic transcripts in LL were detected by JTK [41] at an FDR threshold of 0.6, showing that RAIN is more sensitive than JTK in the case of this data and that both algorithms identify a similar set of transcripts (electronic supplementary material, table S17).

**Figure 2.**
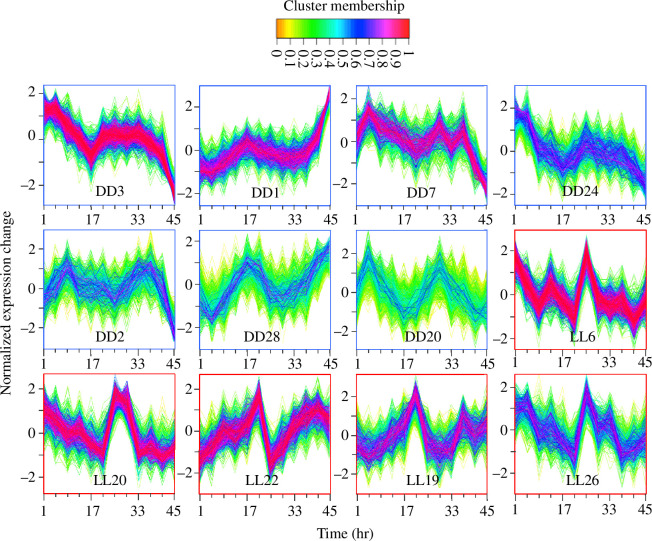
Normalized expression of clusters with significant (*q *< 0.01) over-representations of rhythmic genes. Each transcript profile in each cluster is coloured by that gene’s membership of the cluster.

To test whether the rhythmic transcripts identified in RAIN were over-represented in any particular Mfuzz cluster, I employed hypergeometric tests. Clusters found to have an over-representation of rhythmic transcripts (*q *< 0.05; electronic supplementary material, tables S1 and S2) are shown in figure 2.

**Figure 3.**
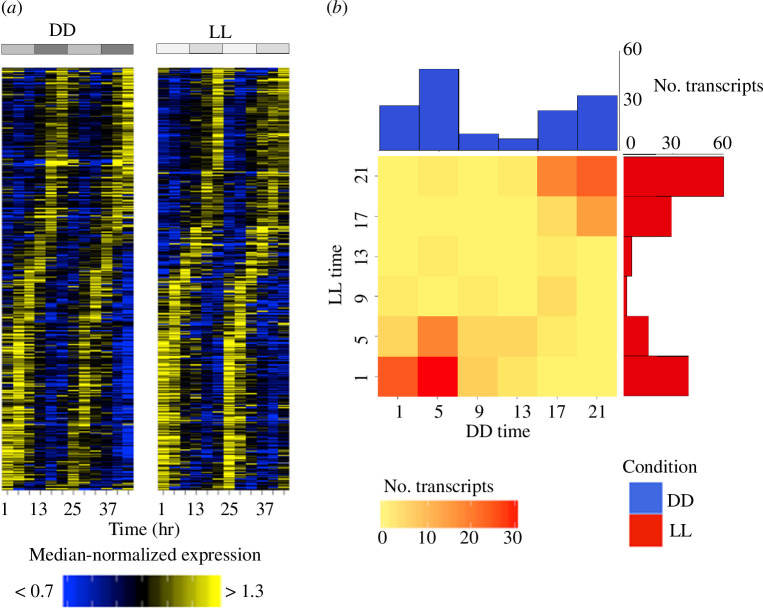
Circadian transcriptional rhythms. (*a*) Heatmap of median-normalized expression of rhythmic (*q *< 0.1) transcripts in both constant darkness and constant light. (*b*) Histograms and heatmap of phases of rhythmic transcripts (*q *< 0.1 in both conditions), showing bimodal phase distribution and overlap between the two conditions.

Rhythmic transcripts (*q *< 0.1) were sorted by phase, peak shape and significance, and plotted (figure 3*a*). Examining the phase distribution (figure 3*b*), it is apparent that the majority of transcripts show peak expression early in the subjective morning or in the subjective night, with fewer transcripts peaking at intermediate times. This trend is also apparent in the phases reported by JTK cycle (electronic supplementary material, figure S3), and so is not algorithm specific. This disparity in phase is greater in the transcripts that show rhythmic expression in both DD and LL; <12% of transcripts in DD and <5% in LL show peak expression at intermediate times (figure 3*b*). The majority of these transcripts (approx. 87%) exhibit a similar (±4 h) phase in LL to their phase in DD.

Similarly to *Drosophila* [42] and mammals [9], the majority of transcripts show only small cyclic changes in expression amplitude over the day; over 80% of reliably quantified (see §4) transcripts in both conditions have amplitudes (peak expression divided by trough expression) of 2 or less. In both DD and LL, transcripts with exceptionally high amplitudes (>4) are transcripts with unusually low or high measurements at isolated time points with no obvious specific shared function. This trend is also visible in the JTK cycle rhythmic transcripts; JTK reports that all reliably quantified cycling transcripts cycle with a median-normalized amplitude of <0.6, and more than 90% of transcripts cycle with a median-normalized amplitude of <0.2 (electronic supplementary material, figure S4). This is in contrast with results in *Drosophila* and mammals, where some core clock genes exhibit very-high-amplitude oscillations [9,42,43].

### (c) Canonical clock genes and comparison with *Drosophila*

The canonical clock genes were examined for rhythmicity both at the transcript level and via an additional RAIN analysis at the gene level. The *q*-values for the canonical clock genes are shown in electronic supplementary material, table S3, along with their expression levels across LL and DD in electronic supplementary material, figure S5. I found rather limited evidence for rhythmicity in these genes that included *Pdp1e* (*q *~ 0.1, LL and DD), *Shaggy* (*q* < 0.1, DD) and *Clock* (*q* ~ 0.1, LL). At a less stringent FDR (*q *< 0.2), *per*, *cyc*, *dbt* and *cwo* were rhythmic in DD, while *cry* and *cyc* oscillate in LL.

I compared the transcripts identified as cycling in *Nasonia* heads with the transcripts identified as cycling in *Drosophila* heads. For these purposes, I used a list of genes identified in a meta-analysis study of *Drosophila* circadian microarray data as being rhythmically expressed in either LD (12 : 12 light : dark) or DD [44]. Of 173 genes identified as rhythmic in *Drosophila*, 33 genes (electronic supplementary material, table S5) were found to also be rhythmic in *Nasonia* (either in LL or DD, *q *< 0.1), no more than would be expected by chance (*p* = 0.11, hypergeometric test).

Additionally, the rhythmic genes were compared with a study examining the *Nasonia* photoperiodic response [45]. Four genes were found to be rhythmic in either DD or LL and also differentially methylated in long and short photoperiods. These genes are the UPF0430 protein CG31712, inositol hexakisphosphate and diphosphoinositol-pentakisphosphate kinase, carboxy terminal domain RNA polymerase II polypeptide A small phosphatase 1 and the endoplasmin gene.

### (d) Functions of rhythmic genes

**Figure 4.**
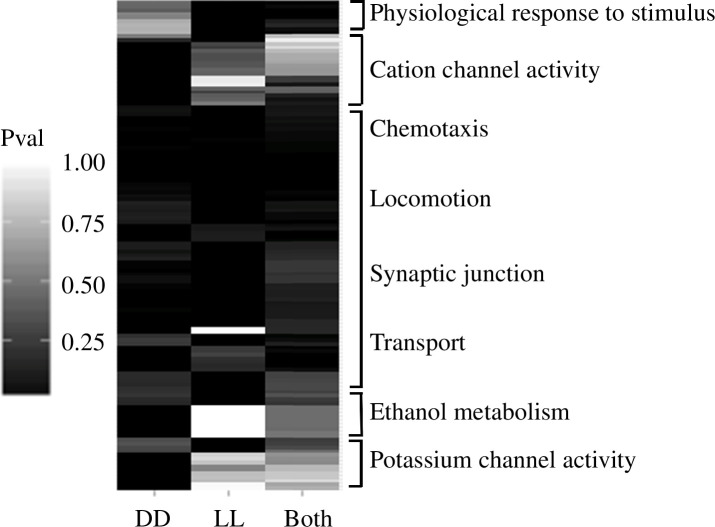
Hierarchical clustering of hypergeometric *p*-values for terms significantly over-represented in at least one condition, showing terms represented in DD-only cycling genes (*q* < 0.2, left column), LL-only cycling genes (*q* < 0.2, middle column) and genes cycling in both conditions (*q* < 0.2, right column).

To capture the general functions that rhythmic genes may fulfil in *Nasonia*, I tested a broader set of rhythmic genes (FDR < 0.2 in RAIN) for GO term over-representation [46,47], revealing 93 GO terms over-represented in genes rhythmic in DD (including ‘response to light stimulus’, ‘proteasome complex’ and ‘generation of neurons’; electronic supplementary material, table S6) and 122 terms for genes rhythmic in LL (including ‘locomotion’, ‘proteasome complex’ and ‘response to external stimulus’; electronic supplementary material, table S7). Hierarchical clustering of hypergeometric *p*-values (figure 4) does not reveal any clear distinction between DD and LL in terms of over-represented functions, with differences between the two conditions primarily consisting of terms with few associated genes (i.e. liable to disruption by noise). From this analysis, it appears that genes cycling in DD and LL fulfil similar functions. Significantly over-represented GO terms shared between both conditions include those related to neurons, signal transmission and responses to stimuli. Notably, all four *Nasonia* opsins were found to exhibit similar transcriptional profiles in LL and DD, with low expression in the morning and high expression in the evening (electronic supplementary material, figure S6).

It has previously been demonstrated that the timing of different (or indeed opposing) biological processes can be controlled through the circadian regulation of groups of genes [48,49]. Unsupervised clustering methods have previously been established as a useful method for the functional characterization of circadian genes [50]. Here, I employed expression clustering analysis to establish whether the temporal separation of functions occurs in *Nasonia*. Examples of clusters with enriched functions include clusters DD7 and LL20, which are significantly enriched for catalytic activity GO terms, especially genes involved in the proteasome, and clusters DD24 and LL6, which are both involved in circadian and neural processes. Other clusters (DD1 and DD2) did not turn up any over-represented GO terms and are thus likely comprised of genes with a wide range of functions.

### (e) Transcriptional differences between constant darkness and constant light

To examine whether differences in circadian period seen in locomotor activity between DD and LL could also be detected in transcriptional rhythms, I fitted parametric models with a range of periods to transcripts rhythmic in both conditions (*q* < 0.1). For those transcripts with statistically significant fits to the model in both conditions (*q* < 0.1; see §4), I took the period with the best fit and compared these periods between conditions. Overall, transcripts in LL showed a significantly (*p* < 3.9 × 10^−9^, Wilcoxon rank-sum test) shorter (median 24) period than those in DD (median 25.4), mirroring the behavioural differences in the period.

**Figure 5.**
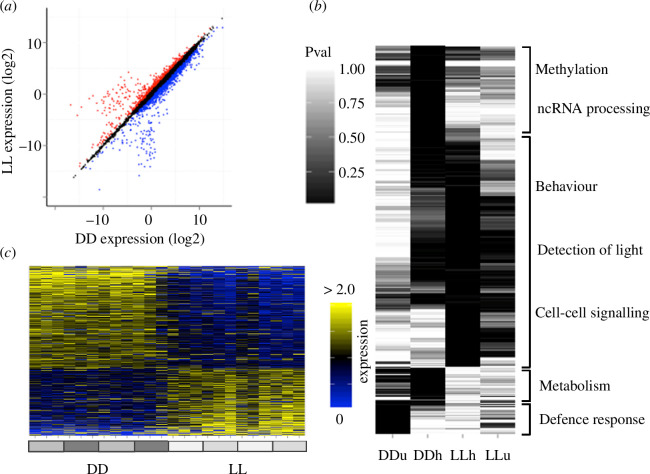
Differential gene expression between DD and LL. (*a*) FPKM (log_2_) expression of transcripts in DD (*x*-axis) and LL (*y*-axis), showing genes classified (>1.5 median fold change) as differentially expressed up in DD (blue) and up in LL (red). (*b*) Hierarchical clustering of hypergeometric *p*-values for terms significantly over-represented in at least one condition. Columns are: DDu (>1.5 fold-change higher in DD), DDh (higher in DD but ≤1.5 fold-change), LLh (higher in LL but ≤1.5 fold-change) and Llu (>1.5 fold-change higher in LL). (*c*) Heatmap showing median-normalized expression for differentially expressed transcripts, in DD (left) and in LL (right), sorted by fold change.

I also tested for differential expression between DD and LL. Due to the lack of biological replicates, I analysed differential expression using a fold-change approach. I used 1.5 fold-change as a cut-off for differential expression [51], yielding 1488 genes expressed higher in DD than in LL and 971 genes expressed higher in LL than in DD (figure 5). Genes more highly expressed in DD were significantly enriched (*q* < 0.01) for various forms of catalytic activity (electronic supplementary material, table S10), including the vast majority of proteasome genes (>75%). Genes more highly expressed in LL were enriched for a small number of terms including ‘plasmalemma’ and ‘sequence-specific DNA binding’ (electronic supplementary material, table S11). Hierarchical clustering of *p*-values (figure 5*b*) shows this distinction more clearly, showing that metabolism and defence response are over-represented in genes more highly expressed in DD, and that terms involved in the detection of light and cell–cell signalling are over-represented in genes more highly expressed in LL.

## 3. Discussion

This study provides the first insights into global transcriptional oscillation in *Nasonia*. With RNA-seq, I profiled the circadian transcription of >26 000 transcripts in *Nasonia* in either DD or LL. At a relatively stringent FDR (*q* < 0.1), I identified 1057 cycling transcripts in DD and 929 cycling transcripts in LL. These transcripts correspond to a cycling fraction of 6.7% and 5.9% of all transcripts analysed in DD and LL, respectively. These figures are comparable to cycling fractions reported in various organisms and tissues, generally between 2% and 10% of the transcriptome [52]. This is the first study to examine the circadian transcriptome in *Nasonia,* as previous studies (e.g. [53,54]) have focused on a few genes likely to be involved in circadian function.

In both conditions, cycling transcripts were found to cycle at low amplitudes (mostly <2-fold) and with a limited, bimodal range of phases. This is in contrast to microarray/RNA-seq studies in *Drosophila*, where transcripts were found to cycle with a broader range of phases [55], and studies in both mammals and *Drosophila*, which have identified a group of high-amplitude (>4-fold) cycling genes among the low-amplitude majority [56]. High-amplitude cyclers typically include clock genes [42,56]. The low cycling amplitudes of CCGs in *Nasonia* may be due to cycling in a relatively small proportion of cells in the *Nasonia* head; however, this does not explain why no differences are observed between core clock genes and other CCGs (i.e. why there are no high-amplitude cyclers even on a relative level). In *Drosophila*, much of this amplitude difference is applied post-transcriptionally, as demonstrated by nascent-seq [55]. The low cycling amplitudes in *Nasonia* could therefore imply differences in post-transcriptional regulation of CCGs. The low oscillations of the *Nasonia* head transcriptome render the expression profiles of the canonical clock genes difficult to resolve, as demonstrated through the comparison of plant microarrays by Covington *et al*. [57]. This issue may also contribute to the discordance between the various circadian microarray studies in *Drosophila* [44].

A major limitation of the current study is using whole head tissue rather than just the brain. Consequently, the data here represent gene expression in both the brain and the compound eyes (a substantial part of the head tissue). The technical ease of using the heads compared to brain dissection allows sampling a larger number of individuals, but at the cost of reduced resolution, which may be the reason for many core clock genes that were not found to be rhythmic. Our study aligns with earlier global expression studies in *Drosophila* that have used whole heads [10,58–60] and were able to capture the oscillating expression of these core genes. In *Nasonia*, whole heads RNA was used to demonstrate the oscillating transcript of *per* and cry [53,61]. Nevertheless, future studies should focus on the brain or even on specific cells in the brain, mirroring the recent methodological developments in *Drosophila* research [42,62,63]. In addition, future research should include a proteomic survey, as it is possible that core clock proteins maintain rhythmicity due to post-transcriptional/translational mechanisms despite non-rhythmic mRNA.

An emerging characteristic of the circadian transcriptome in *Nasonia* is the temporal separation of function by phase (figure 4). Notably, genes involved in catalytic activity were strongly over-represented in morning-peaking transcripts. This is in line with other studies that show catalytic activity confined to the morning in fungi [48], in agreement with a general observation that an important (or even primary) function of circadian clocks [64] is to temporally separate catabolism and anabolism. Although I did not detect an over-representation of anabolic genes within the cyclic transcripts, expression clusters DD10 and LL24 (electronic supplementary material, figures S1 and S2) did show strong over-representation (electronic supplementary material, tables S12 and S13) for genes involved in cytosolic ribosomal genes (*q* < 3 × 10^−56^) and cellular anabolism (*q* < 2 × 10^−6^). These clusters exhibit an antagonistic expression pattern to the expression clusters containing the catabolic genes, suggesting that catabolism and anabolism are indeed separated by the circadian clock in *Nasonia*.

Though our analysis consisted of two independent experiments, the comparison of expression between LL and DD reveals that a majority of genes involved in the proteasome and a broader set of genes involved in catabolism were more highly expressed in DD than in LL. As turnover rates of clock proteins have shown to be coupled with changes in the circadian period [65,66], pending confirmation, upregulation of the proteasome may provide an explanation for differences in the period observed between DD and LL.

The genes that cycle in *Drosophila* largely differ from those cycling in *Nasonia*. These differences in CCG identity between *Drosophila* and *Nasonia* may represent significant biological distinctions, demonstrating the plasticity in which genes can be rhythmically transcribed to regulate specific functions in a circadian manner. Examples of functions shared by CCGs in the *Drosophila* and *Nasonia* heads include various aspects of metabolism [55,58–60], phototransduction [55,59], synaptic/nervous functions [10,58,60], oxidoreductase activity [58], mating behaviour [55] and immunity [10,60].

## 4. Methods

### (a) Maintenance and sample collection

Stocks of *N. vitripennis* (strain AsymCX) were maintained at 25°C on blowfly pupal hosts in 12 : 12 light : dark cycles. To obtain male wasps for experiments, groups of eight females were isolated at the yellow pupal stage and transferred onto fresh hosts upon eclosion. The resulting male progeny were collected upon eclosion and moved onto vials with a 30% sucrose agar medium, in groups of 20. During entrainment (four full days in a 12 : 12 light : dark cycle) and collection, wasps were kept in four LED light boxes in the same incubator at 19°C. Starting at CT1, wasps were collected every 4 h over a period of 48 h (in either DD or LL), and snap-frozen in liquid nitrogen and immediately transferred to −80°C. This sampling approach was chosen to prioritize high temporal resolution, which reduces temporal variance and strengthens the ability to detect oscillatory patterns in gene expression. Given the nature of rhythmic analysis, increasing the number of sampling points within each cycle is statistically more beneficial for detecting periodicity than additional biological replicates, which would increase biological variance without improving rhythmic detection power.

Wasps were collected sequentially from light box to light box every 4 h to minimize disturbance of wasps, and so wasps were collected from each light box once every 16 h, thereby minimizing the effect of variations within light boxes. Light intensity recordings showed inter-light-box variation of 6–21 lum ft^−2^ during the entrainment period of DD and 0–13 lum ft^−2^ during the entrainment and constant period of LL. Given these variations, I verified that wasps entrained correctly to the experimental conditions and that free-running behaviour was as expected. Individual male wasps were isolated, and locomotor activity was monitored matching the experimental conditions, both in light intensity and temperature. Behavioural recordings of individual male wasps in experimental conditions can be seen in electronic supplementary material, figure S7, ruling out behavioural differences caused by inter-light-box variations in light intensity in LL, though not necessarily transcriptional differences.

### (b) RNA extraction, sequencing and read mapping

RNA was extracted from pooled groups of 50 heads for each sample, using Trizol RNA extraction protocol, and followed by clean-up using the RNAeasy spin column kit (Qiagen). Samples were polyA selected and sequenced at Glasgow Polyomics (University of Glasgow, United Kingdom) on the Illumina NextSeq500 platform, resulting in approximately 20 million 75 bp paired-end reads per sample.

Read mapping was achieved with Tophat2 (v2.1.0) [67] against the *Nasonia* Nvit_2.1 NCBI annotation. As the purpose of this study was not to identify novel splice variants or improve on existing annotation, novel junction detection was disabled for accurate quantification of known transcripts. Mean mapping efficiency was above 90% for both conditions (electronic supplementary material, table S14). Read quantification was performed using the DEseq normalization method [68]. All 24 samples from both conditions were grouped to allow comparison between and within conditions.

### (c) Expression profile clustering

Isoform expression profiles were first filtered to include only those isoforms with no missing values at any time point in either condition. Expression values were standardized using the ‘Standardize’ function in Mfuzz [38]. The ‘cselection’ function in Mfuzz was used to select an appropriate c-value for the c-means clustering (default parameters; *m* = 1.25). Based on this analysis, 30 fuzzy clusters were generated for each condition using the fuzzification parameter *m* = 1.25.

### (d) Rhythmic expression analysis

RAIN [40] was used on all filtered isoforms (i.e. those with no missing values at any time point) in either condition to detect rhythmic isoforms at a period of 24 h. As a non-parametric method, RAIN only facilitates detection of rhythmic isoforms with periods that are a multiple of the sample resolution (in this case 4 h). The *p*-values produced by RAIN were corrected to *q*-values using the Benjamini–Hochberg method [69]. This method was repeated using expression values for genes rather than transcripts for the clock gene analysis (i.e. the summed expression values for all known transcripts of a particular gene).

Maximum fold changes in expression were calculated by normalizing per-condition expression values by the median value and calculating the ratio from the lowest expression over 48 h to the highest. Reliably quantified transcripts are defined as those transcripts where the absolute FPKM value is 5 or above at all time points, the threshold for this set at a similar level to other analyses [42].

To analyse the period of rhythmic transcripts, I fitted parametric waveforms with a variety of periods (20–28 h in steps of 0.2 h) to all transcripts identified as rhythmic (*q* < 0.1) in both conditions. This FDR threshold is in line with, or more strict than, thresholds chosen in other similar studies [42,44,70]. Those transcripts (85 in total) that showed a significant (*q* < 0.1) fit to the model in both conditions were analysed in terms of their best fitting period.

GO term over-representation was performed in WaspAtlas [46] using the Nvit_2.1 NCBI annotation dataset. All hypergeometric tests were performed within R using the ‘phyper’ function. Clusters with rhythmic components were identified by collapsing the fuzzy clusters into hard clusters using the ‘cluster’ property of the Mfuzz object, performing hypergeometric tests to identify clusters with enrichment for rhythmic transcripts. Thirty tests were performed for each condition (i.e. for all clusters) and were corrected per-condition using the Benjamini–Hochberg method implemented in the R software [71].

For comparison to microarray studies, orthologues for *Drosophila melanogaster* were obtained from a meta-study of circadian microarray data [44]. The 214 obtained FlyBase identifiers were converted to the latest identifiers using the validation tool, resulting in 218 unique identifiers (the increase in identifiers can be attributed to previous identifiers referring to multiple genes in the current annotation). Orthologues for these *Drosophila* genes were obtained through WaspAtlas, retrieving orthologues for 135 genes that mapped to 173 unique *Nasonia* genes due to gene duplications, etc. This set of 173 genes was compared with the number of genes with rhythmic transcripts that would be expected by chance using a hypergeometric test.

Hierarchical clustering of hypergeometric *p*-values was performed in R using the hclust function. For each GO term significantly over-represented in at least one condition, the absolute difference between *p*-values (within each condition) was summed over all conditions. This was used as the distance metric for clustering. GO terms were sorted based on the results of the clustering and presented using ggplot. A summary of the entire pipeline can be found in the electronic supplementary material.

## Data Availability

I have made the expression profile for each transcript in both conditions available on WaspAtlas [46]. Data have been archived in the NCBI short read archive (SRA), with accession number PRJNA318159. Supplementary material is available online [72].
